# Developmental Assets and Their Relationship to Suicidal Behavior in Mexican Young Adults

**DOI:** 10.3390/ijerph21081068

**Published:** 2024-08-15

**Authors:** Omar Baza-Arce, Angélica Juárez-Loya, Catalina González-Forteza

**Affiliations:** 1 Clinical and Health Psychology Department, Psychology Faculty, Universidad Nacional Autónoma de México (UNAM), Mexico City 04510, Mexico; psicomarbaza@gmail.com; 2 Epidemiology and Psychosocial Research Department, Instituto Nacional de Psiquiatría Ramón de la Fuente Muñiz, Mexico City 14370, Mexico; catiartes@gmail.com

**Keywords:** suicidal behavior, positive youth development (PYD) framework, developmental assets, young students

## Abstract

Suicide in young people is a public health problem. Typically, protective factors for suicide are not studied; research tends to focus on measuring risk factors. However, knowing the risk factors does not mean that we also know the opposing factors that influence a group’s health problems. For this reason, we examined the relationship between developmental assets in Mexican youth aged 18 to 25 years who are not at risk for suicide, exhibit self-injurious behavior, and whose last suicide attempt had low or high lethality. A cross-sectional study of 478 young people (73% female and 27% male) from Mexico City was conducted using an online survey and correlations were tested with dummy variables (groups) and multinomial logistic regression. The no-risk group showed associations with all developmental assets, the self-injurious group had an association with the house rules variable, the low lethality group was correlated with twelve assets and the high lethality group with four assets. Four internal developmental strengths were significant in the regression model: avoidance of risk behaviors, school expectations, resistance to pressure, and expression of anger. These results suggest that PYD is a useful framework for examining suicide risk and promotes skill development in young college students.

## 1. Introduction

Suicide among young people is a worrying problem. Worldwide it was the fourth leading cause of death among 15–29 year olds in 2019 [1]. The 2019 Global Burden of Disease (GBD) study [2] shows that self-harm is the third leading cause of disability among people aged 10–24.

According to the Instituto Nacional de Estadística y Geografía (INEGI) (National Institute of Statistics and Geography) [3], at least 1800 deaths by suicide among people aged 15 to 24 are recorded annually in Mexico between 2018 and 2021. In 2022, 2002 people in this age group died (861 people aged 15 to 19 and 1141 people aged 20 to 24).

It is important to examine the factors associated with suicide and the behaviors that precede it (e.g., self-injurious behavior, suicidal ideation, or suicide attempts). There is a lack of information on the factors that protect adolescents and young adults from developing these behaviors. In general, research examines the risk factors for suicide, but this does not necessarily tell us about the strategies that might prevent it.

The Positive Youth Development (PYD) framework states that developmental assets promote improvement in social, academic, or vocational skills and lead to improved well-being and good mental health [4]. This model originates from community [5] and positive psychology [6].

According to the PYD framework (a) youth who realize their full potential are less likely to have problems, (b) support and opportunities are important for youth success, (c) communities can improve their capacity to build successful youth, and (d) youth should be seen as people who need to be nurtured and developed, not as people with problems [7].

The Search Institute’s Developmental Assets Framework is one of the most widely used and influential frameworks for PYD. It proposes 40 developmental assets, half of which are internal and the other half external [8].

External assets were defined as experiences and opportunities in family, school, youth programs, and communities that promote youth resilience and thriving. These were grouped into four categories: support (and acceptance by others), empowerment (in terms of feeling valued and valuable, feeling safe and respected), boundaries and expectations (because young people need clear rules, consistent consequences for breaking rules, and encouragement to do their best), and constructive use of time (meaning they deserve to develop new skills and interests outside of school) [8].

Internal assets are personal skills, values and self-perceptions that help young people make good choices, take responsibility for their own lives, be independent and thrive. They are grouped into four categories: commitment to learning (a sense of the enduring importance of learning and recognition of their own abilities), positive values (principles that help them make healthy life choices), social competencies (skills to interact effectively with others, make difficult decisions, and deal with new situations), and a positive identity (young people need to believe in their own self-worth and feel that they have control over the things that happen to them) [8].

Regarding suicide, developmental assets were mainly studied in adolescents (aged 12 to 19 years). As expected, some authors reported that the ability to make decisions, general self-confidence, general hope for the future, religiosity, good health practices, family communication, parental supervision, positive relationship with mother, appropriate time management, connectedness to school, commitment to community [9], commitment to learning, positive values, social skills, positive identity, support, self-determination, clarification of boundaries, and set expectations protected them from sadness [10], suicidal ideation [9], and suicide attempts [10].

In another study, developmental assets were associated with academic stress, suicide risk and emotional regulation self-efficacy. Controlling by gender, developmental assets moderated the relationship between emotional regulation self-efficacy and suicide risk. In addition, higher levels of developmental assets limited the indirect effects of academic stress on suicide risk through emotional regulation self-efficacy [11].

In a Mexican study, two groups of adolescents aged 15 to 18 years (with and without suicide attempt) were compared to examine differences between developmental strengths. They found differences between external assets (mother’s support and supervision and father’s support and supervision) and internal assets (importance to health, avoidance of risk behaviors, and decision making) [12].

Therefore, we set out to examine the relationship between developmental assets and risk level of last suicide attempt in Mexican youths aged 18 to 21 years. We wanted to investigate the protective factors that should be promoted to ideally contribute to a reduction in suicide prevalence. To this end, we compared youth without a history of suicidal behavior (who were classified as not suicidal) with youth who had experienced three types of suicide attempts: (a) self-injurious behavior, in which individuals expressed a desire to live despite the attempt; (b) low-lethality suicide attempts, in which individuals reported hurting themselves without a clear intention to die (ambiguity); and (c) high-lethality suicide attempts, in which individuals had a clear desire to die but did not accomplish it. This conceptualization of suicidal behavior is supported by research that has identified four latent classes [13,14,15] in the analysis of suicidal behavior and associated psychosocial factors among adolescents in Campeche, Guanajuato and Querétaro (Mexico).

## 2. Materials and Methods

### 2.1. Study Design, Setting, and Population

A cross-sectional study was conducted in which 478 young people (73% female and 27% male) from Mexico City with an average age of 21 years (*SD* = 1.88) were studied. Most of the participants attended college (89.3%), 6.5% attended high school, and the rest had another degree (4%). Sampling was defined as non-probabilistic and intentional. Given the 554,990 college students enrolled in Mexico City for 2020–2021 [16], we applied a 95 confidence level, a 5% margin of error, and a 50 population proportion to the formula. This resulted in 384 participants. However, given the low control conditions of this sample, we decided to study at least 500 individuals.

### 2.2. Constructs and Measurements

#### 2.2.1. Internal and External Assets for Adolescents (FIE-AR)

The test consisted of 65 Likert-type items with 5 response options assessing 14 developmental assets, 6 external (mother’s supervision, mother’s support, father’s supervision and support, house rules, friends without risk behavior, and healthy friends) and 8 internal (responsibility, importance of health, avoidance of risk behaviors, resistance to pressure, importance of religion, decision making, expression of anger, and school expectations). The confirmatory factor analysis yielded a model that was a good fit with the external (*X*^2^ = 3326.14, *degrees of freedom* = 1013, Root Mean Squared Error of Approximation (RMSEA) = 0.039, 95% Confidence Interval (CI) (0.038–0.041), Comparative Fit Index (CFI) = 0.983) and internal assets (*X*^2^ = 1491.63, *df* = 499, RMSEA = 0.037, 95% CI (0.034–0.039), CFI = 0.986). In addition, the Cronbach’s alphas for the external assets were between 0.62 and 0.96 for the subscales and between 0.65 and 0.93 for the internal assets [17].

#### 2.2.2. Suicidal Behaviors Schedule (CCS)

The Suicidal Behaviors Schedule (Cédula de Conductas Suicidas) was used as a screening instrument to determine differences between self-harm and low or high risk of a last suicide attempt (called lethality). The first questions investigated whether participants had self-harmed, cut or poisoned themselves in order to take their own lives. They were also asked about the number of suicide attempts, age at first and last attempt, purpose, motivation, methods used, and use of mental health services (in the form of open-ended questions), as well as an indicator of the lethality of these behaviors (i.e., the desire to die by suicide). Combining the data on suicidal behavior with the indicator of the degree of risk of the last suicide attempt (classified by the desire to die) provides information to better understand and characterize the type of single or last suicidal behavior (categorized as no-risk, self-injurious behavior, low-lethality suicide attempt, or high-lethality suicide attempt) [13,18].

Numerous studies with Mexican adolescents have shown that the questionnaire has concordant validity with variables such as depression, drug use and impulsivity and divergent validity with family relationships, self-esteem, and internal locus of control in relation to suicidality [13,18,19].

### 2.3. Data Collection and Analysis Procedure

Data were collected using an electronic questionnaire via Google forms (free version, Google LLC, Mountain View, CA, USA). Participants were invited via the college’s social networks in the first quarter of 2022. The only requirements were that they were studying at the time of answering the survey, lived in Mexico City, and were between 18 and 25 years old (601 young people answered the questionnaire, but the cases that did not meet the criteria described were removed from the database).

Taking into account the Declaration of Helsinki [20], participants were informed via a consent form about the conditions of their participation and the handling of the data collected (no personal data were requested) and also received information about low-cost mental health centers for young people.

The program Jamovi (version 2.3.28, The Jamovi Project, Newcastle, Australia) [21] was used to standardize the values of the scales (with the intention of allowing a fair comparison between the values of the scales involved), and to examine the data with descriptive statistics, we created dummy variables with suicide risk categories to test associations with developmental assets through Spearman’s rho test. In this way, we can determine which developmental assets are most strongly associated with each risk group. Finally, a multinomial logistic regression model was conducted to assess the no-risk group versus the self-injurious behavior group, the no-risk group versus the low-lethality suicide attempt group, and the no-risk group versus the high-lethality suicide attempt group. We considered that the no-risk group gave us a better understanding of the developmental assets that could prevent suicidal behavior.

## 3. Results

Table 1 shows the proportion of cases analyzed according to the degree of suicide attempt risk and gender. Most of the sample was not at risk of suicide, and 27% of the sample was at some risk.

The group correlation test (dummy variables) showed mostly positive associations with all developmental assets for the no-risk group, an inverse association was only found for the variables friends without risky behavior (external asset), resistance to pressure and expression of anger (internal asset). The group with self-injurious behavior only showed an inverse correlation with the variable house rules (external asset). The low-lethality suicide attempt group showed an inverse correlation with mother’s supervision and support, father’s supervision and support (external assets), and six internal assets: responsibility, importance of health, avoidance of risk behaviors, importance of religion, decision making, and school expectations. In addition, this group showed a direct correlation with resistance to pressure and expression of anger (both internal assets). The high-lethality suicide attempt group showed an inverse correlation with the external assets: mother’s support and father’s supervision and support, and with the internal assets: importance of health and school expectations (Table 2).

The multinomial logistic regression model for the comparison of the group without risk with the group with self-injurious behavior yielded the following results: the variable avoidance of risk behavior (internal asset) was a protective factor, meaning that an increase in the standard deviation of this variable was associated with a lower probability of belonging to the group with self-injurious behavior (OR = 0.534; 95% CI = 0.290–0.983); whereas the variable expression of anger (internal asset) was a risk factor, indicating that an increase in the standard deviation of this variable was associated with a higher likelihood of belonging to the self-injurious behavior group (OR = 2.233; 95% CI = 1.185–4.209) (Table 3).

In the comparison of the no-risk group with the low-lethality suicide attempt group, only the variable of anger expression (internal asset) was found to be a risk factor, indicating that an increase in the standard deviation of this variable was associated with a higher probability of belonging to the low-lethality group (OR = 2.233; 95% CI = 1.185–4.209) (Table 3).

When comparing the no-risk group with the group who attempted suicide with a high lethality, the resistance to pressure variable (internal asset) proved to be a risk factor, i.e., an increase in the standard deviation of this variable was associated with a higher probability of belonging to the group with high lethality (OR = 1.698; 95% CI = 1.083–2.662). In contrast, the variable school expectations (internal asset) proved to be a protective factor, as an increase in the standard deviation of this variable was associated with a lower probability of belonging to the high lethality group (OR = 1.698; 95% CI = 1.083–2.662). See Table 3.

## 4. Discussion

Suicide in young people needs to be looked at from different angles. The PYD framework [8] gave us some positive and alternative explanations that promote well-being and mental health in young people. The main idea behind this study was to investigate the relationship between developmental assets and risk level of last suicide attempt in Mexican youth students (18–21 years old).

In the correlation analysis with dummy variables by group, the no-risk group showed positive associations with most developmental assets.

Among the external development assets, the quality of parental supervision and support were positively associated with belonging to this group, as was the presence of rules at home. Parental supervision and support were also inversely related to belonging to the low and high-lethality suicide attempt groups, confirming that the lower the quality of the relationship with parents, the greater the likelihood of belonging to these two groups. An inverse relationship was observed between home rules and the self-injurious behavior group, suggesting that the fewer rules there are at home, the higher the likelihood of self-harm.

In the previous literature on risk factors for suicide in adolescents, parental relationship and supervision play a protective role in suicidal behavior [9,10,22], but also in other problems such as substance use and depression [10,15,22]. Another study on college students, which examined the family environment (socioeconomic level, parents’ relationship, parents’ level of education, parents’ occupation and number of siblings), went in the same direction. The participants with suicidal ideation showed similarities in that their family suffered from poverty, had poor family relationships, parents had job insecurity, and may have had an inappropriate parenting style [23].

In terms of friends, the analysis showed that the perception of having healthy friends and having more friends without risky behavior (this category is reverse scored on the scale, i.e., a lower score means that the characteristic is more pronounced) are associated with belonging to the no-risk group. No correlation was found between the variables of friends and risk groups. Some studies have shown that suicidality is lower in youth with stronger social ties to peers, family or other adults. These relationships affect psychological well-being [24], increase help-seeking [25], and promote adaptive coping [26]. One of the internal developmental assets identified in association with the non-risk group was responsibility. As expected, the greater the responsibility, the greater the likelihood of belonging to the no-risk group. This variable was also significant, but negative, in the low-lethality suicide attempt group, i.e., individuals who were undecided about their desire to die but made an attempt anyway, a behavior that could be explained by impulsivity or low responsibility for oneself. One study has shown that responsible decision making reduces the likelihood of suicidal ideation in young people. Interventions to promote responsible decision making, good health practices and general self-awareness can improve young people’s overall mental health [9].

Avoidance of risk behaviors (internal asset) was significantly associated with the no risk behaviors group (positive association) and with the low lethality group (negative association), and in the regression model, greater avoidance of risk behaviors led to a lower likelihood of belonging to the self-injurious behavior group (as opposed to the no risk behaviors group). This confirms that young people who make responsible choices and are self-aware, as mentioned by Lensch et al. [9], are more likely to be healthy.

Importance to health was positively related to the no-risk group and negatively related to the groups with low and high lethality. This result is consistent with the study by Lensch et al. [9], in which health practices were a protective factor for suicidal ideation.

Decision making was positively related to the no-risk group and negatively related to the low-lethality group but was not significant in the regression model. As Lensch et al. [9] report, this is an important skill for young people and appears to be an important topic for health promotion.

Another internal asset that was negatively associated with the no-risk group was resistance to pressure. It was also positively associated with the low-lethality group and was seen to be a risk factor for being in the high-lethality suicide attempts group in the regression model. It seems that young people who endure more than a certain level of pressure learn to tolerate an undesirable level of social stress, which probably leads to emotional stress and, in combination with other factors, to a higher risk of suicidal behavior. Wiium et al. [10] reported in their study that resistance to pressure is a social skill and they observed more competencies in groups without persistent sadness and no suicide attempts.

The importance of religion had a positive correlation with the no-risk group, a negative correlation with the low-risk group, and no significance in the regression model. Previous research had reported that religiosity is a protective factor against suicide [27], but our results did not show this.

In the Spearman test, the variable expression of anger showed a negative association with the no-risk group, a positive association with the low-lethality group, and a null association with the high-lethality group. There was also a risk factor in the regression model for self-injurious behavior and the low-lethality groups. This information is consistent with the literature, which has reported that increased anger is associated with a higher risk of suicide [11,28,29]. Indeed, the literature reports that inadequate anger management is a primary factor influencing self-injurious behavior [30].

School expectations were positively related to the no-risk group, negatively related to the low- and high-lethality groups, and found to be a protective factor for the high-lethality group. Previous research had reported in a recent meta-analysis [31] and in studies by Lensch et al. [9] that school connectedness is a protective factor against suicidal ideation and behavior.

All developmental assets were associated with the no-risk group, confirming their importance for better youth development. The results of this study shed light on the Search Institute’s Developmental Assets Framework [8] in young adults and show that they are as important in young students as they are in adolescence.

The findings on external developmental assets (parents’ supervision and support and house rules) confirm that the family environment needs to be proactive and supportive in order to provide young people with a sense of autonomy, responsibility and skill, while providing adult support and supervision. Regarding the role of friends, it is important to collect more data and explore suicide prevention programs that target peer relationships. These types of interventions could help young people strengthen their bonds, seek support and expand their peer networks. An example of this could be the Guardians program, where peers gain the skills to recognize early signs of suicide risk, assess the level of risk, and have motivational conversations with at-risk individuals. They can also refer people to appropriate screening and treatment [32].

Only the internal development assets were significant in the regression model. Risk avoidance behavior and school expectations were protective for the self-injurious behavior group and for the high-lethality group. In contrast, anger expression (significant for the self-injurious behavior and low lethality groups) and resistance to pressure (significant for the high lethality group) were risk factors.

These data suggest that school continues to play an indispensable role in the well-being of young people. To the extent that they are empowered in emotion regulation and decision making, this may also have an impact on suicide risk. Suicide prevention and mental health promotion programs can be implemented in schools. Examples of prevention programs in school settings have shown that students who benefit from these programs are less likely to experience depressive episodes, suicidal ideation, and suicide attempts than other schools that do not benefit from these programs [33].

### Limitations

Due to the nature of the data (cross-sectional measurement and not a representative sample of the college population), the results should be interpreted with caution. However, this study provides a picture of the importance of developmental assets and their role in relation to youth who reported having attempted suicide for various reasons. It is advisable to replicate the study, but now selecting a representative sample of the university population and trying to achieve a balance between men and women and to increase the number of individuals reporting different levels of risk. With a sample that has these characteristics, it would be possible to draw more reliable conclusions.

## 5. Conclusions

We hypothesized that developmental assets would be protective factors for suicide risk in young Mexican college students. As expected, all developmental assets in the correlation test were associated with the no suicide risk group and some of them had theoretical agreement with the groups categorized as young people with self-injurious behavior and with the low and high-lethality suicide attempt risk groups.

According to the logistic regression model evaluated in this sample, four internal developmental strengths were significant: avoidance of risk behaviors, school expectations, resistance to pressure, and expression of anger. These aspects have been associated with suicidal behavior [9,10,11,28,29,30,31], and we can recommend based on this research that these aspects can be implemented in intervention and prevention programs for suicidal behavior in young students.

These interventions should focus on helping youth strengthen their relationship with educational institutions and view school as a safe environment in which they can grow academically and personally.

Emotional dysregulation should also be at the center of any intervention to impact suicide risk. This includes life skills training, such as decision making to avoid risky practices, or promoting functional coping strategies to reduce tolerance to pressure and deal appropriately with stressful situations they are exposed to.

It is also recommended to measure representative samples of this population and promote applied research to evaluate the impact of suicide prevention programs in Mexican youth, with developmental assets that can determine the objectives of these types of programs.

The PYD framework is a valid theory and requires further scientific evidence in the Mexican context for the prevention of different risk factors in adolescence and youth. It is recommended to develop a more complete psychometric instrument that includes 40 developmental assets. It is also important to further investigate suicide risk using the PYD framework.

## Figures and Tables

**Table 1:** Descriptive outcomes by suicide risk levels and sex.

	Counts		% of Total		
Suicide Risk Levels	Women	Men	Women	Men	% of Total
High-lethality suicide attempt	30	7	6.3%	1.5%	7.8%
Low-lethality suicide attempt	56	18	11.8%	3.8%	15.6%
Self-injurious behavior	16	1	3.4%	0.2%	3.6%
No-risk	245	103	51.5%	21.5%	73.0%

**Table 2:** Correlation Matrix by Suicide risk levels.

Variable	1	2	3	4	5	6	7	8	9	10	11	12	13	14	15	16	17	18
1. No-risk group	—																	
2. Self-injurious behavior group	**−0.317**	—																
3. Low-lethality suicide attempt group	**−0.707**	−0.083	—															
4. High-lethality suicide attempt group	**−0.479**	−0.056	**−0.125**	—														
5. Mother’s supervision ◆	**0.213**	−0.043	**−0.206**	−0.043	—													
6. Mother’s support ◆	**0.216**	−0.015	**−0.178**	**−0.108**	**0.689**	—												
7. Father’s supervision and support ◆	**0.225**	−0.062	**−0.128**	**−0.16**	**0.307**	**0.406**	—											
8. House rules ◆	**0.096**	**−0.095**	−0.062	−0.008	**0.298**	**0.287**	**0.216**	—										
9. Friends without risky behavior ◆	**−0.129**	0.089	0.064	0.064	**−0.171**	−0.002	−0.074	**−0.104**	—									
10. Healthy friends ◆	**0.126**	−0.063	−0.084	−0.05	**0.217**	**0.262**	**0.183**	**0.132**	−0.028	—								
11. Responsibility ◆	**0.162**	0.014	**−0.158**	−0.065	**0.294**	**0.232**	**0.204**	**0.124**	−0.067	**0.242**	—							
12. Importance of health ◆	**0.273**	−0.023	**−0.232**	**−0.122**	**0.305**	**0.353**	**0.258**	**0.14**	−0.023	**0.293**	**0.349**	—						
13. Avoidance of risk behaviors ◆	**0.196**	−0.066	**−0.183**	−0.03	**0.352**	**0.221**	**0.196**	**0.139**	**−0.361**	**0.094**	**0.224**	**0.315**	—					
14. Resistance to pressure ◆	**−0.132**	0.032	**0.133**	0.016	**−0.232**	**−0.12**	−0.054	−0.035	**0.245**	−0.047	**−0.154**	**−0.102**	**−0.275**	—				
15. Importance of religion ◆	**0.096**	−0.003	**−0.093**	−0.032	**0.221**	**0.268**	**0.201**	**0.261**	**−0.116**	0.046	**0.126**	**0.272**	**0.263**	**−0.1**	—			
16. Decision making ◆	**0.202**	−0.053	**−0.156**	−0.087	**0.21**	**0.225**	**0.256**	0.03	−0.013	**0.154**	**0.282**	**0.312**	**0.224**	**−0.165**	−0.007	—		
17. Expression of anger ◆	**−0.204**	0.088	**0.204**	0	−0.064	−0.044	**−0.125**	0.013	**0.142**	**−0.12**	**−0.092**	**−0.095**	−0.026	0.054	0.052	**−0.124**	—	
18. School expectations ◆	**0.234**	−0.047	**−0.16**	**−0.139**	**0.236**	**0.228**	**0.201**	0.069	0.015	**0.134**	**0.417**	**0.373**	**0.131**	−0.08	**0.098**	**0.304**	−0.063	—

◆ Denotes a continuous, standardized variable with mean = 0 and standard deviation = 1; bold numbers indicate a confidence interval of *p* ≤ 0.05 in Spearman’s rho test.

**Table 3:** Results of multinomial logistic regression for suicide lethality risk and development assets.

	No-Risk vs. Self-Injurious Behavior				No-Risk vs. Low-Lethality				No-Risk vs. High-Lethality			
	95% CI				95% CI				95% CI			
Predictor	Estimate	OR	Lower	Upper	Estimate	OR	Lower	Upper	Estimate	OR	Lower	Upper
Intercept	**−4.042**	0.018	0.006	0.052	**−1.874**	0.154	0.104	0.226	**−3.248**	0.039	0.018	0.083
Mother’s supervision ◆	0.007	1.007	0.385	2.637	−0.104	0.901	0.565	1.436	0.246	1.278	0.634	2.576
Mother’s support ◆	0.014	1.014	0.375	2.739	−0.215	0.807	0.492	1.323	−0.059	0.943	0.466	1.907
Father’s supervision and support ◆	−0.260	0.771	0.348	1.710	0.024	1.025	0.675	1.555	−0.646	0.524	0.265	1.036
House rules ◆	−0.592	0.553	0.255	1.197	−0.078	0.925	0.635	1.347	0.034	1.035	0.595	1.799
Friends without risky behavior ◆	0.308	1.360	0.600	3.086	0.163	1.177	0.770	1.798	0.255	1.291	0.698	2.386
Healthy friends ◆	−0.655	0.520	0.233	1.156	−0.027	0.973	0.661	1.432	−0.102	0.903	0.496	1.645
Responsibility ◆	0.920	2.511	0.995	6.334	0.049	1.050	0.708	1.556	0.476	1.609	0.828	3.125
Importance of health ◆	−0.442	0.643	0.287	1.438	−0.304	0.738	0.497	1.096	−0.551	0.577	0.320	1.039
Avoidance of risk behaviors ◆	**−0.627**	0.534	0.290	0.983	−0.258	0.772	0.529	1.129	−0.158	0.854	0.476	1.532
Resistance to pressure ◆	−0.053	0.948	0.421	2.139	0.224	1.251	0.881	1.775	**0.530**	1.698	1.083	2.662
Importance of religion ◆	0.170	1.185	0.578	2.431	−0.018	0.983	0.656	1.472	0.101	1.106	0.589	2.080
Decision making ◆	−0.046	0.955	0.480	1.899	−0.280	0.756	0.518	1.102	−0.212	0.809	0.447	1.466
Expression of anger ◆	**0.803**	2.233	1.185	4.209	**0.632**	1.881	1.331	2.657	0.008	1.008	0.590	1.724
School expectations ◆	−0.300	0.741	0.341	1.609	−0.330	0.719	0.489	1.057	**−0.927**	0.396	0.218	0.720

◆ Denotes a continuous, standardized variable with mean = 0 and standard deviation = 1; OR denotes “odds ratio”; 95% CI denotes “95% confidence interval”; No-risk = no-risk of suicide; Low-lethality = low-lethality suicide attempt group; High-lethality = high-lethality suicide attempt group; the reference variable was “No-risk group”; bold numbers indicate a confidence interval of *p* ≤ 0.05. Overall model test: *χ*^2^ = 118, *degrees of freedom* = 42, *p* > 0.001.

## Data Availability

Data are not available due to ethical restrictions.
